# Fiber Bragg Grating Pressure Sensor Integrated with Epoxy Diaphragm

**DOI:** 10.3390/s21093199

**Published:** 2021-05-05

**Authors:** Shiuh-Chuan Her, Shin-Zhong Weng

**Affiliations:** Department Mechanical Engineering, Yuan Ze University, Chung-Li 320, Taiwan; s1035026@mail.yzu.edu.tw

**Keywords:** fiber bragg grating, pressure sensor, epoxy diaphragm, sensitivity

## Abstract

A fiber Bragg grating (FBG) sensor integrated with an epoxy diaphragm was developed for the measurement of pressure and water level. The bending strain of a circular diaphragm induced by uniform pressure was transferred to the FBG sensor. The response of the FBG sensor to the pressure was observed in terms of the Bragg wavelength shift which is linearly proportional to the strain. The effect of epoxy diaphragm thickness on the sensitivity and accuracy was investigated. The experimental results show that the sensitivity of FBG/epoxy diaphragm pressure sensor is decreasing with the increase of the diaphragm thickness. The sensitivities of the FBG pressure sensors with diaphragm thicknesses of 0.5 mm, 0.7 mm, and 1.0 mm were 175.5 pm/kPa, 89.5 pm/kPa, and 43.7 pm/kPa, respectively. The pressure measured by the proposed FBG sensor was compared with theoretical prediction and a close agreement was observed.

## 1. Introduction

Pressure is one of the most important parameters for structural safety monitoring in engineering applications. Development of accurate and effective sensors for the measurement of liquid level or pipe pressure are essential in a variety of industries, such as the oil [1] and chemical [2] industries. Various sensing methodologies based on mechanical, electrical and optical techniques have been proposed for the evaluation of pressure and liquid level. Traditional electrical or mechanical pressure sensors have limited capabilities in harsh environments such as series electromagnetic interference [3], high temperature and pressure [4,5], dangerous chemicals, or explosive substances [6]. In addition, single-point measurement and incapable of remote transmission and on-line monitoring further restrict the applications of the traditional electrical and mechanical pressure sensors [7]. Optical finer sensors provide an attractive alternate due to their obvious advantages such as small size, light weight, corrosion resistance, immunity to electromagnetic interference, distributed capability, and remote sensing [5,8,9]. There are three different types of optical fiber pressure sensors: intensity-based [10]; fiber Bragg grating (FBG)-based [11]; and Fabry-Perot cavity based [12].

Recently, FBG pressure sensors have been extensively studied and widely implemented, since their measurements are based on the Bragg wavelength and independent on the light intensity, connector, or fiber loss. Furthermore, FBG sensors can be multiplexed in a single optical fiber to achieve on-line and quasi-distributed monitoring. Zhao et al. [13] proposed an FBG pressure sensor based on the diaphragm-cantilever with a sensitivity of 258.28 pm/MPa in the range of 0–2 MPa. Hong et al. [14] fabricated an FBG pressure sensor using fused deposition modelling process. Leal-Junior et al. [15] developed an FBG sensor embedded in polymer for the simultaneous measurements of temperature and pressure. Zhao et al. [16] presented an integrated FBG sensor for the measurements of pressure, temperature, and flow rate, with resolutions of 6 KPa, 0.1°C, and ${0.17\;m}^{3}/h$, respectively. Stephens et al. [17] developed a FBG pressure sensor for the monitoring of patients with ventricular assist devices. The FBG pressure sensor exhibited a sensitivity of 4.6 pm/mmHg with an accuracy of 0.35 mmHg. Ameen et al. [18] integrated an FBG sensor with a graphene diaphragm for the simultaneous measurements of the water level and temperature with high sensitivities of 24.84 pm/cm and 13.31 pm/°C, respectively. Al-Fakih et al. [19] embedded an FBG sensor in a sensing pad to measure the interface pressure within prosthetic sockets. Gutierrez-Rivera et. al. [20] proposed a low-pressure optical fiber sensor based on a thin polyester film, using a phase signal analysis. Leal-Junior et al. [21] developed a temperature-insensitive FBG pressure sensor with temperature cross-sensitivity of 0.33 Pa/°C in a pressure range of 0–1.2 kPa. Wang et al. [22] integrated a diaphragm-assisted Fabry-Perot interferometer with a fiber Bragg grating for pressure measurement with temperature compensation. Liu et al. [23] employed an FBG pressure sensor to study the penetration mechanism of jacked piles in viscous soil foundation. Cheng et al. [24] developed a highly sensitive gas pressure sensor based on a Fabry–Perot interferometer with a silicone rubber diaphragm.

The pressure sensitivity of a bare FBG sensor is 3.04 pm/MPa [25], which is too low to be employed for the practical applications. To improve the FBG pressure sensitivity, several mechanical systems incorporating FBG sensing technology have been proposed, such as FBG embedded in a polymer [26,27], metal coated FBG on a cylinder [28], and FBG bonded on a triangular cantilever [29]. However, some of the methods involve relatively complicated structures which are not easy to be prepared and multiplexed in single optical fiber. In this work, a simple FBG pressure sensor integrated with a circular epoxy diaphragm was proposed and studied. Epoxy, with favorable mechanical properties, low shrinkage, high thermal and chemical resistance, excellent adhesion to various substrates, has a variety of applications, such as coatings and sealants, repairs, electrical insulators, and adhesives for structural components. Epoxy is also known for its corrosion prevention properties in combination with strong adhesive properties, making it a favorable choice for the diaphragm sensor to monitor the liquid level and hydrostatic pressure. Epoxy exhibits performance advantages over the other polymers (e.g., polyester and vinyl esters) in several areas such as superior adhesive properties, better flexibility, resistance to fatigue and micro cracking, reduced degradation from water ingress, and surface degradation due to water permeability. The pressure sensitivity of the FBG sensor can be improved by integration with the epoxy diaphragm, due to its high flexibility. The bending strain of the diaphragm due to the pressure is transferred to the FBG sensor, resulting in a Bragg wavelength shift. Thus, the FBG pressure sensor measures the strain induced Bragg wavelength shift to correlate with the change of pressure. Experimental results show that the Bragg wavelength shift is linearly proportional to the pressure applied. The performance of the FBG pressure sensor was verified in the laboratory with good accuracy. The effect of the diaphragm thickness on the pressure sensitivity was presented.

## 2. Materials and Methods

### 2.1. FBG Sensing Principle

A fiber Bragg grating was written into the core of an optical fiber using a high energy optical source and a phase mask [10]. The fiber core was exposed to interference fringe pattern of ultraviolet light. An in-line optical grating with periodically refractive index was fabricated. When a broadband light travels through the Bragg grating, a specific narrow band was reflected from the grating and all other wavelengths were transmitted. The reflected wavelength known as Bragg wavelength $\lambda_{B}$ was dependent on the effective refractive index $n_{eff}$ and grating period Λ as follow.

(1) $$\lambda_{B}=2n_{eff}\Lambda$$

A shift of the Bragg wavelength $\Delta \lambda_{B}$ due to the modulation of effective refractive index $n_{eff}$ and grating period Λ caused by the external strain ε and temperature change ΔT is given by [15]

(2) $$\Delta \lambda_{B}=\left[\left(1-p_{e}\right)\varepsilon +\left(\alpha +\zeta \right)\Delta T\right]\lambda_{B}$$

where $p_{e}=0.22$ is the strain-optic constant [30], α and ζ are the thermal expansion coefficient and thermal-optic coefficient of the optic fiber, respectively.

The FBG sensing principle is based on Equation (2). Utilizing Equation (2), the external strain ε and temperature change ΔT can be evaluated by measuring the Bragg wavelength shift $\Delta \lambda_{B}$. FBG sensors have been widely used in the field of structural heath monitoring through the measurements of the strain and temperature.

In the case of constant temperature ΔT=0, the Bragg wavelength shift as shown in Equation (2) due to an applied strain can be simplified as

(3) $$\Delta \lambda_{B}=\lambda_{B}\left(1-p_{e}\right)\varepsilon$$

### 2.2. Design and Fabrication of FBG Pressure Sensor

**Figure 1:**
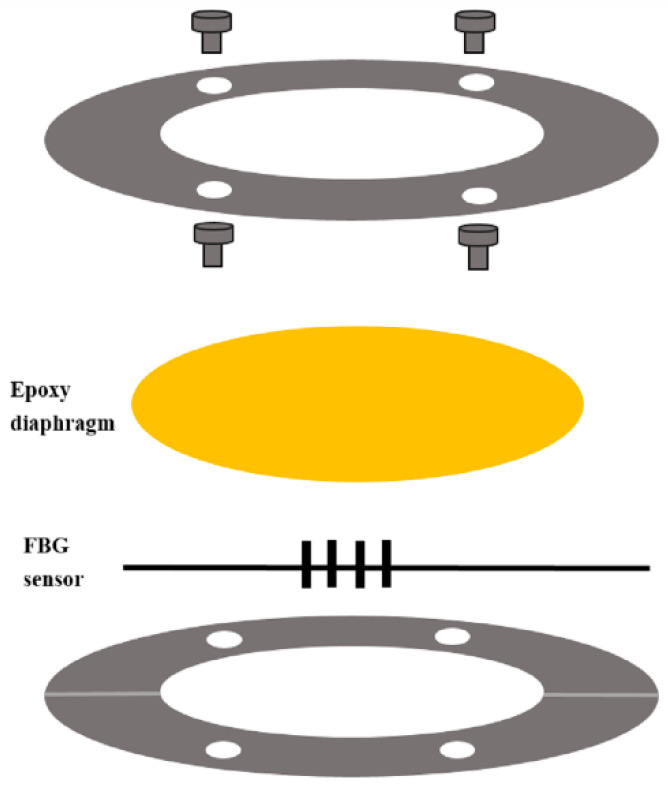
Schematic diagram of fiber Bragg grating (FBG) pressure sensor.

A schematic diagram of the basic structure of FBG pressure sensor is depicted in Figure 1. The sensing structure consists of a circular diaphragm, two annular plates, and a FBG sensor. The circular diaphragm with a diameter of 50 mm was made of epoxy through a spinning coating process. In this work, a low viscosity epoxy Mungo 4200A part A and curing agent 4200B part B provided by Uchess Co. (New Taipei City, Taiwan) were used. The weight ratio between the epoxy and curing agent was 2:1 as recommended by the manufacturer. The curing agent was added into the liquid epoxy, and slowly stirred for 10 min. Then, the mixture was degassed in a vacuum chamber at room temperature for 15 min to remove trapped air induced by the stirring [31]. The degassed mixture was poured onto a circular acrylic plate with a diameter of 50 mm and placed in a spinning coating machine (RMT-SC 150SS, Reliable-Mate Technology Co. Ltd., Shin-Chu City, Taiwan). The thickness of the epoxy diaphragm can be modulated by varying the rotating speed of the spinning coating. After completion of the spinning coating process, the epoxy diaphragm was kept on the acrylic plate at room temperature for 24 h to allow the curing of the epoxy. The prepared epoxy was generally nonreactive, stable, resistant to water, and flexible. The annular plate is made of acrylic material with inner and outer radii of 30 mm and 80 mm, respectively. There are four holes with a diameter of 4 mm and a groove in the acrylic plate as shown in Figure 1. The FBG sensor was adhered to the center of the epoxy diaphragm. The epoxy diaphragm was placed between the two annular acrylic plates and fastened by four screws.

**Figure 2:**
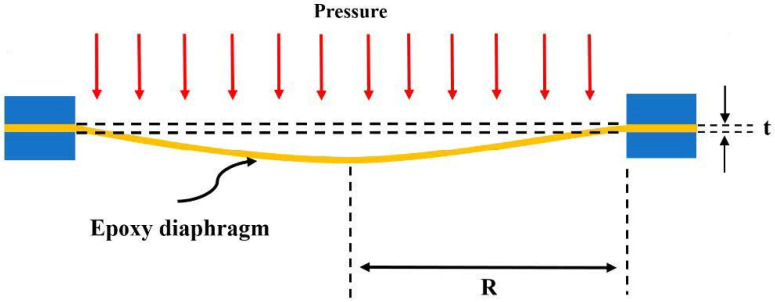
Deformation of a circular diaphragm subjected to an uniform pressure.

The circular diaphragm was clamped and subjected to an uniform pressure as shown in Figure 2. The tangential and radial strains in a circular diaphragm bent by an uniform pressure and clamped along its edge are as follows [32].

(4) $$\varepsilon_{t}=\frac{3p}{8Et^{2}}\left(1-\upsilon^{2}\right)\left(R^{2}-r^{2}\right)$$

(5) $$\varepsilon_{r}=\frac{3p}{8Et^{2}}\left(1-\upsilon^{2}\right)\left(R^{2}-3r^{2}\right)$$

where p is the uniform pressure, E, t, υ and R are the Young’s modulus, thickness, Poisson’s ratio, and radius of the circular diaphragm, respectively; *r* is the distance measured from the center of the circular diaphragm.

The FBG sensor was bonded at the center of the circular diaphragm along the radial direction. The strain was transferred from the diaphragm through the adhesive and protective coating of the optical fiber to the FBG senor. It is well known that bonding characteristics such as the adhesive, bonding length, and protective coating of the optical fiber can affect the strain transfer between the epoxy diaphragm and FBG sensor. In this work, the FGB sensor was adhered to the diaphragm using epoxy. Epoxy with excellent adhesive properties lead to strong bonding between the FBG sensor and diaphragm. Thus, strain can be transferred from the diaphragm to the FBG sensor. The radial strain of the circular diaphragm induced by the uniform pressure is transferred to the FBG sensor as follows

(6) $${\left(\varepsilon_{r}\right)}_{r=0}=\frac{3p}{8Et^{2}}\left(1-\upsilon^{2}\right)R^{2}$$

where E, υ, R, t are the Young’s modulus, Poisson’s ratio, radius and thickness of the diaphragm, respectively.

The Bragg wavelength shift of the FBG sensor due to the uniform pressure can be obtained by substituting the strain from Equation (6) into Equation (3), which yields

(7) $$\Delta \lambda_{B}=\lambda_{B}\left(1-p_{e}\right)\frac{3p}{8Et^{2}}\left(1-\upsilon^{2}\right)R^{2}$$

Thus, the pressure can be determined through the measurement of the Bragg wavelength shift using the following relationship.

(8) $$P=\frac{\Delta \lambda_{B}}{\lambda_{B}}\frac{8Et^{2}}{3\left(1-p_{e}\right)\left(1-\upsilon^{2}\right)R^{2}}$$

### 2.3. Experimental Setup and Measurements

**Figure 3:**
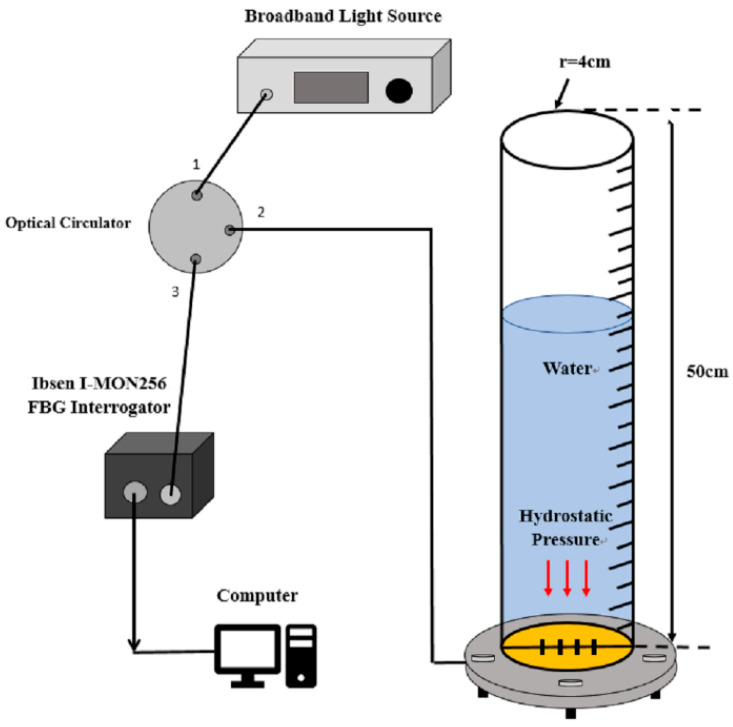
Schematic diagram for the measurements of hydrostatic pressure and liquid level.

**Figure 4:**
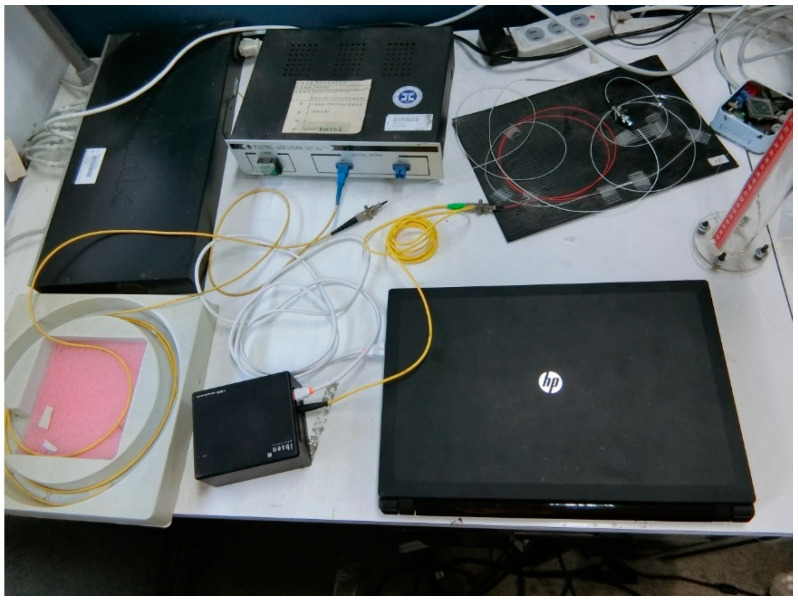
Experimental setup for the hydrostatic pressure and liquid level measurements.

**Figure 5:**
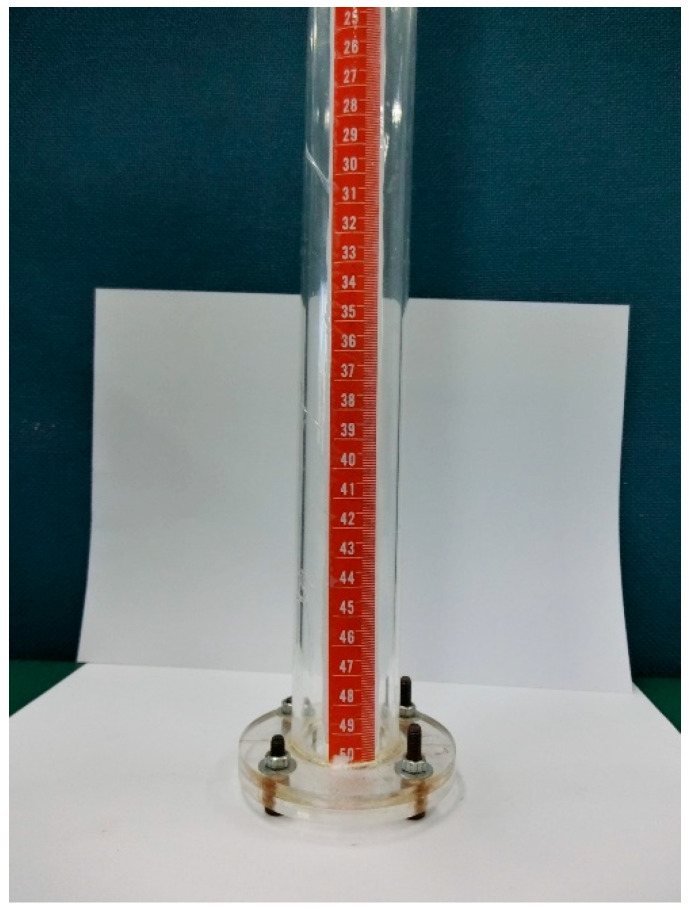
FBG pressure sensor unit installed at the bottom of a liquid container.

Figure 3 illustrates the schematic diagram used to evaluate the performance of the FBG pressure sensor. The sensing system consists of a broadband light source (ASE 1550A, Faztec Co., New Taipei City, Taiwan), a single mode fiber optic circulator (6015-3-APC, Thorlabs Inc., Newton, New Jersey, USA), FBG modular interrogator (I-MON 256, Ibsen Photonics, Farum, Denmark) and the FBG-based sensor unit. The experimental setup is shown in Figure 4. The FBG-based diaphragm sensor was installed at the bottom of a liquid container made of acrylic material with dimensions of diameter 4 cm and length 50 cm as shown in Figure 5. The sensitivity of the FBG pressure sensor is dependent on the deflection of the diaphragm caused by the hydrostatic pressure due to the liquid weight which is a function of the height and density of the liquid, as follows

(9) p=ρgh

where *p* is the hydrostatic pressure, ρ and *h* are the density and height of the liquid, respectively; *g* is gravitational acceleration.

When the FBG-based diaphragm sensor was placed at the bottom of liquid, the hydrostatic pressure induced a compression on the diaphragm resulting in the tangential and radial strains as shown in Equations (4) and (5), respectively. The radial strain caused an extension of the FBG sensor, leading to a Bragg wavelength shift $\Delta \lambda_{B}$ from which the pressure can be determined using Equation (8).

It is well known that FBG sensor is sensitive to both the temperature and strain. In this work, an FBG sensor was placed at the bottom of a cylindrical water container. The water temperature was kept at the room temperature and monitored by a thermocouple. The experimental tests were conducted at the room temperature and maintained the thermal stability with a constant temperature to eliminate the cross influence between the temperature and pressure.

## 3. Results and Discussion

**Figure 6:**
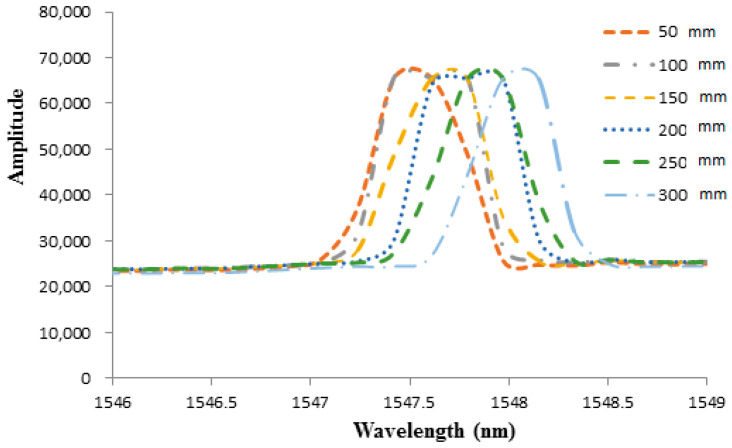
Optical spectra of Bragg wavelength reflected from the FBG sensor under various water levels with diaphragm thickness of 0.5 mm.

**Figure 7:**
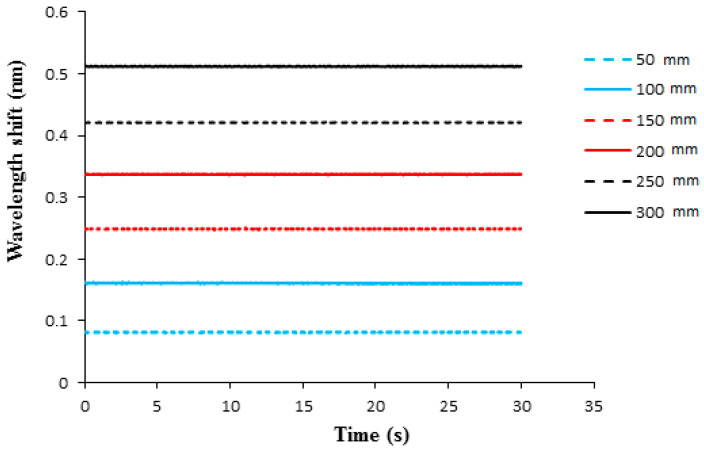
Bragg wavelength shift from the FBG sensor under various water levels with diaphragm thickness of 0.5 mm.

Liquid weight exerted hydrostatic pressure on the surface of the epoxy diaphragm lead to deformation and induced a strain to the adhered FBG sensor. The strain of the FBG sensor caused a Bragg wavelength shift, which can be demodulated by a FBG interrogator. In this work, the FBG sensitivity against the pressure is evaluated in terms of Bragg wavelength shift as the water level is increased from 5 cm to 30 cm in a step of 5 cm, which corresponds to a hydrostatic pressure increases from 490.5 Pa to 2943 Pa with an increment of 490.5 Pa. The thickness of the epoxy diaphragm is 0.5 mm. The optical spectra of Bragg wavelength reflected from the FBG sensor under various water levels recorded by an optical spectrum analyzer (AQ 6331, Ando Electric Co., Tokyo, Japan) are plotted in Figure 6. The Bragg wavelength shifts measured by FBG interrogator with water level varying from 5 cm to 30 cm are shown in Figure 7. It can be observed that the Bragg wavelength shift increased with the increase of the water level. The pressure can be determined by substituting the Bragg wavelength shift $\Delta \lambda_{B}$ into Equation (8). The Young’s modulus and Poisson’s ratio of the epoxy are 1.99 GPa and 0.3, respectively. The radius and thickness of the epoxy diaphragm were 15 mm and 0.5 mm, respectively. The Bragg wavelength $\lambda_{B}$ was 1547.245 nm. The experimental measurements of the hydrostatic pressure by the FBG sensor are validated with the theoretical prediction Equation (9), as shown in Table 1. It appears that the measured error of the FBG pressure sensor decreased with the increase of the water level. The maximum error occurred at the water level of 5 cm, with an error of 5.59%. Meanwhile, the pressure measured by the FBG sensor at the water level of 30 cm was significantly reduced to an error of 0.83%. This demonstrates that the proposed FBG sensor is capable of measuring the pressure with a good accuracy.

**Figure 8:**
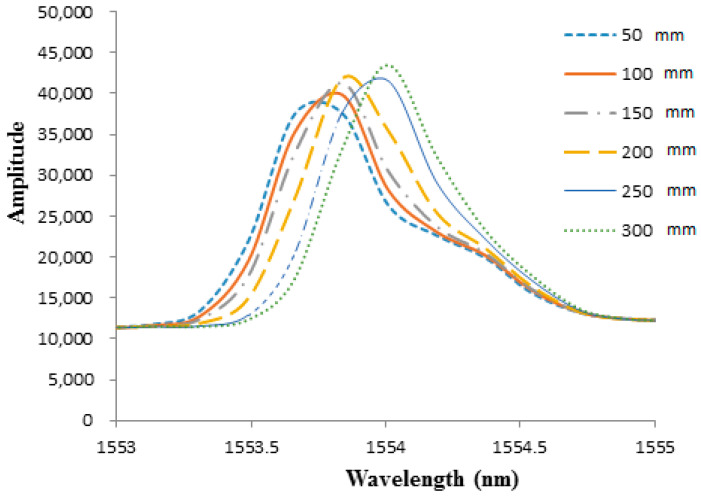
Optical spectra of Bragg wavelength reflected from the FBG sensor under various water levels with diaphragm thickness of 0.7 mm.

**Figure 9:**
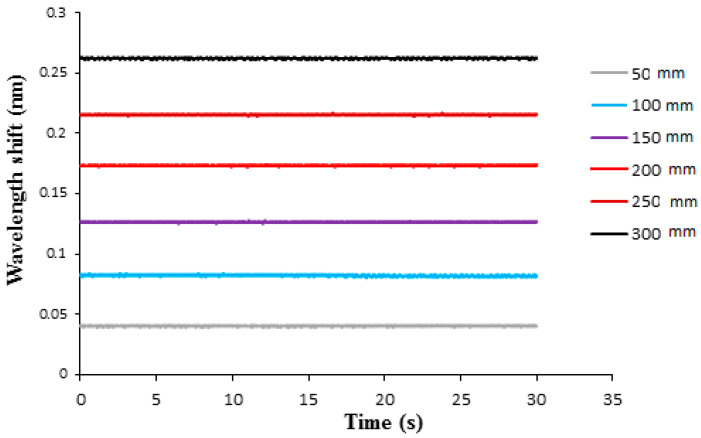
Bragg wavelength shift from the FBG sensor under various water levels with diaphragm thickness of 0.7 mm.

**Figure 10:**
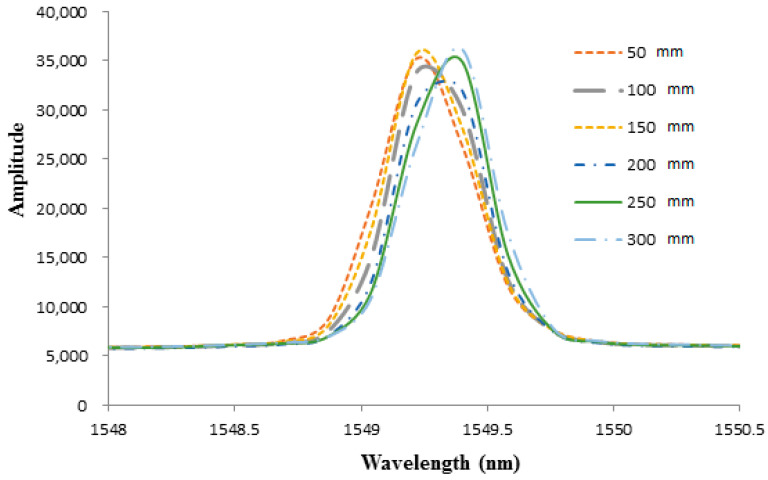
Optical spectra of Bragg wavelength reflected from the FBG sensor under various water levels with diaphragm thickness of 1.0 mm.

**Figure 11:**
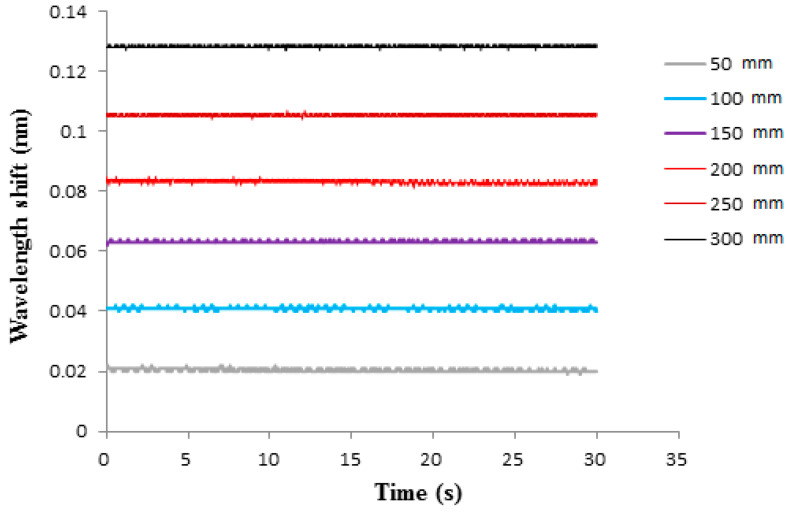
Bragg wavelength shift from the FBG sensor under various water levels with diaphragm thickness of 1.0 mm.

To investigate the effect of diaphragm thickness on the pressure sensitivity of the FBG sensor, two epoxy diaphragms with thicknesses of 0.7 mm and 1.0 mm were prepared. The optical spectra of the Bragg wavelength reflected from the FBG sensor bonded on the epoxy diaphragm with thicknesses of 0.7 mm and 1.0 mm are illustrated in Figure 8 and Figure 9, respectively. The Bragg wavelength shifts for diaphragm thicknesses of 0.7 mm and 1.0 mm are presented in Figure 10 and Figure 11, respectively. The hydrostatic pressures under various water levels measured by the FBG sensor with diaphragm thicknesses of 0.7 mm and 1.0 mm are presented in Table 2 and Table 3, respectively. The hydrostatic pressures measured by the FBG sensor are compared with theoretical prediction Equation (9). Experimental results for diaphragm thicknesses of 0.7 mm and 1.0 mm are similar with the thickness of 0.5 mm. The accuracy increased with the increase of the water level, i.e., the higher pressure the better the accuracy that was achieved. Moreover, the accuracy of the FBG sensor was also dependent on the diaphragm thickness. The accuracy can be improved by decreasing the diaphragm thickness. The measured errors at water level 30 cm for diaphragm thicknesses of 0.5 mm, 0.7 mm, and 1.0 mm were 0.83%, 0.94%, and 1.35%, respectively.

In this work, the Bragg wavelength was detected by a FBG interrogator (I-MON 256, Ibsen Photonics) with a resolution of 0.5 pm in the range of 1525 nm ~ 1570 nm. The errors of the FBG pressure sensor with epoxy diaphragm thicknesses of 0.5 mm, 0.7 mm, and 1.0 mm were 2.86 Pa, 5.5 Pa, and 11.4 Pa, respectively. The error of the proposed FBG pressure sensor can be attributed to the measurement error of the Bragg wavelength by the FBG interrogator (I-MON 256). The strain of the diaphragm induced by the hydrostatic pressure increased with the decrease of the diaphragm thickness, as shown in Equation (6). The Bragg wavelength shift of the FBG sensor exhibited the same trend as the diaphragm strain, i.e., increasing with the decrease of the diaphragm thickness. The measurement error of the FBG pressure sensor due to the wavelength measured error from the FBG interrogator can be calculated as follows

(10) $$\mathrm{Er}=\frac{\lambda_{error}}{\Delta \lambda_{B}}$$

where $\lambda_{error}=0.5\;\mathrm{pm}$ denotes the wavelength measured error from the FBG interrogator; $\Delta \lambda_{B}$ is the Bragg wavelength shift.

The Bragg wavelength shift increased with the decrease of the diaphragm thickness, leading to a decrease of the measurement error.

**Figure 12:**
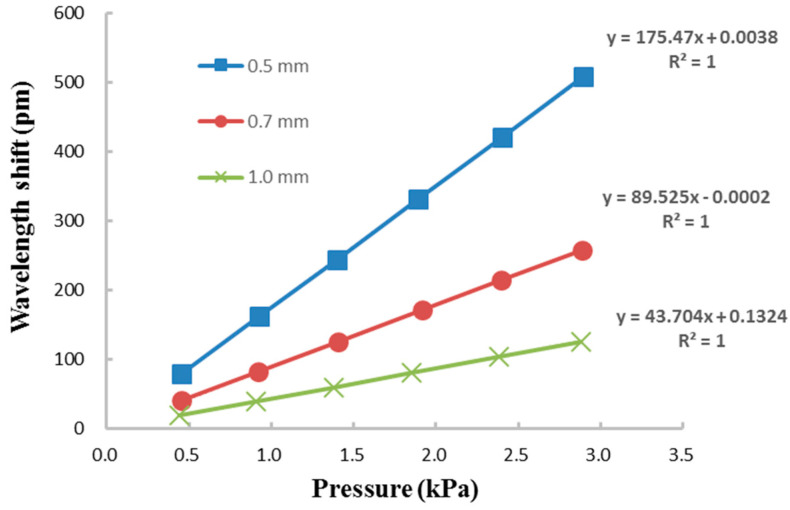
Linear relationship between the Bragg wavelength shift and pressure with different diaphragm thicknesses of 0.5 mm, 0.7 mm and 1.0 mm.

The relationship between the Bragg wavelength shift and the pressure for diaphragm thicknesses of 0.5 mm, 0.7 mm, and 1.0 mm are plotted in Figure 12. It can be seen that the Bragg wavelength shift is linearly proportional to the pressure. The pressure sensitivity of the FBG sensor is defined as

(11) $$\mathrm{ps}=\frac{\Delta \lambda_{B}}{\Delta p}$$

where $\Delta \lambda_{B}$ and Δp are the Bragg wavelength shift and pressure change, respectively.

The pressure sensitivity of the FBG sensor can be extracted from the curve of the linear relationship between the Bragg wavelength shift and pressure as shown in Figure 12. Experimental results depict that the pressure sensitivities of the FBG sensor with diaphragm thicknesses of 0.5 mm, 0.7 mm, and 1.0 mm are 175.5 pm/kPa, 89.5 pm/kPa, and 43.7 pm/kPa, respectively. The pressure sensitivities of FBG sensors integrated with metal diaphragm and polymer diaphragm reported by Pachava et al. [33] and Ahmad et al. [26] were 32.0 pm/kPa and 8.7 pm/kPa, respectively. It shows that the pressure sensitivity 175.5 pm/kPa of the proposed FBG sensor integrated with epoxy diaphragm is better than that of metal [33] and polymer [26] diaphragms. The Bragg wavelength shift increased with the decreases of the Young’s modulus, and the thickness of the diaphragm as well as the increase of the radius, as shown in Equation (6). Pressure sensitivity can be enhanced by increasing the Bragg wavelength shift. Pachava et al. [33] developed a FBG pressure sensor using aluminum diaphragm. The proposed FBG sensor integrated with an epoxy diaphragm is better than that of aluminum diaphragm [33] due to a lower Young’s modulus of epoxy in comparison with aluminum. Ahmad et al. [26] prepared a FBG sensor embedded in a polymer diaphragm with thickness and radius of 4 mm and 10 mm, respectively. A thinner thickness in conjunction with a larger radius of the epoxy diaphragm used in this work attributes to a higher sensitivity than that of the FBG sensor by Ahmad et al. [26]. The proposed FBG sensor can be employed to monitor the liquid level with sensitivities of 16.2 pm/cm, 8.2 pm/cm, and 4.2 pm/cm for water while integrated with the epoxy diaphragm with thicknesses of 0.5 mm, 0.7 mm, and 1.0 mm, respectively. It can be observed that the pressure sensitivity of the FBG sensor increased with the decrease of the diaphragm thickness. The high sensitivity of the diaphragm integrated FBG sensor with a thinner thickness is attributed to its high flexibility during the deformation induced by the hydrostatic pressure. The water level sensitivities of FBG sensors integrated with carbon fiber composite diaphragm and silicone rubber diaphragm reported by Song et al. [32] and Marques et al. [34] were 1.85 pm/cm and 10.2 pm/cm, respectively. The water level sensitivity 16.2 pm/cm of the proposed FBG sensor is better than that of the FBG sensor reported by Song et al. [32], owing to a lower Young’s modulus of the epoxy compared with the carbon fiber composite. Furthermore, the water level sensitivity of the proposed FBG sensor with a thinner diaphragm thickness of 0.5 mm is better than that of FBG sensor reported by Marques et al. [34], with a thicker diaphragm thickness of 1.1 mm.

Diaz et al. [35] developed a water level sensor consisting of two FBGs, one of the FBG embedded into epoxy diaphragm the other FBG served as a temperature reference sensor. The sensing system provided temperature compensation to improve the accuracy of liquid level measurement. In this work, single FBG was employed and attached on the surface of the epoxy diaphragm. The strain transferred from the epoxy diaphragm to the embedded FBG sensor is more effectively than that of surface bonded FBG sensor, resulting in a higher water level sensitivity of 27.4 pm/cm [35]. However, the fabrication process of surface bonded FBG on an epoxy diaphragm is much easier than embedding FBG into epoxy. In addition, a theoretical model was presented to analyze the pressure induced Bragg wavelength shift which is related to the water level. The proposed FBG pressure sensor was experimentally conducted and verified by the theoretical prediction with very good accuracy. Moreover, a highly linear response to the pressure with linear correlation coefficient greater than 99% was observed. The comparisons of the pressure and water level sensitivities between the proposed FBG sensor and the other FBG sensors reported in the literature were presented in Table 4.

## 4. Conclusions

In this work, an epoxy diaphragm integrated FBG sensor was developed to measure the pressure and liquid level. The feasibility of the proposed FBG sensor has been verified by the theoretical prediction with excellent correlation. The effect of diaphragm thickness on the pressure sensitivity was investigated. Experimental results show that the pressure sensitivity of the FBG sensor increased with the decrease of the diaphragm thickness. The pressure sensitivity of the FBG sensor with diaphragm thicknesses of 0.5 mm, 0.7 mm, and 1.0 mm were 175.5 pm/kPa, 89.5 pm/kPa, and 43.7 pm/kPa, respectively. This demonstrates that the proposed FBG sensor exhibits high sensitivity with good accuracy. Moreover, the FBG sensor has features of small size, low cost, simple structure, easy fabrication and installation. It has a good prospect in practical applications.

## Figures and Tables

**Table 1:** Experimental measurement and theoretical prediction of hydrostatic pressure under various water levels with diaphragm thickness of 0.5 mm.

Water Level (mm)	Theoretical Prediction Equation (9) (Pa)	$\mathrm{Bragg}\;\mathrm{Wavelength}\;\mathrm{Shift}\;\Delta \lambda_{B}\;(\mathrm{nm})$	Experimental Measurement Equation (8) (Pa)	Error
50	491	0.081	463.53	5.59%
100	981	0.163	932.78	4.92%
150	1472	0.25	1430.65	2.81%
200	1962	0.337	1928.51	1.71%
250	2453	0.422	2414.93	1.55%
300	2943	0.51	2918.52	0.83%

**Table 2:** Experimental measurement and theoretical prediction of hydrostatic pressure under various water levels with diaphragm thickness of 0.7 mm.

Water Level (mm)	Theoretical Prediction Equation (9) (Pa)	$\mathrm{Bragg}\;\mathrm{Wavelength}\;\mathrm{Shift}\;\Delta \lambda_{B}\;(\mathrm{nm})$	Experimental Measurement Equation (8) (Pa)	Error
50	491	0.0412	460.21	6.27%
100	981	0.0826	922.65	5.95%
150	1472	0.129	1440.94	2.11%
200	1962	0.173	1932.42	1.51%
250	2453	0.216	2412.73	1.64%
300	2943	0.261	2915.39	0.94%

**Table 3:** Experimental measurement and theoretical prediction of hydrostatic pressure under various water levels with diaphragm thickness of 1.0 mm.

Water Level (mm)	Theoretical Prediction Equation (9) (Pa)	$\mathrm{Bragg}\;\mathrm{Wavelength}\;\mathrm{Shift}\;\Delta \lambda_{B}\;(\mathrm{nm})$	Experimental Measurement Equation (8) (Pa)	Error
50	491	0.02	457.21	6.88%
100	981	0.041	937.28	4.46%
150	1472	0.062	1417.35	3.71%
200	1962	0.083	1897.42	3.29%
250	2453	0.1053	2407.21	1.87%
300	2943	0.127	2903.28	1.35%

**Table 4:** Comparisons of the pressure and water level sensitivities between the present and other FBG sensors reported in the literatures.

Pressure Sensitivity		Water Level Sensitivity	
Present	175.5 pm/kPa	Present	16.2 pm/cm
Pachava et al. [33]	32.0 pm/kPa	Diaz et al. [35]	27.4 pm/cm
Ahmad et al. [26]	8.7 pm/kPa	Marques et al. [34]	10.2 pm/cm
Liang et al. [7]	0.34 pm/kPa	Song et al. [32]	1.85 pm/cm

## Data Availability

Data available on request.
